# Prenatal Diagnosis of Vasa Previa in the Second Trimester of Pregnancy Based on Non-typical Ultrasound Findings: A Case Report and Mini-Review of the Literature

**DOI:** 10.7759/cureus.58575

**Published:** 2024-04-19

**Authors:** Efthymia Thanasa, Anna Thanasa, Ioannis-Rafail Antoniou, Ektoras-Evangelos Gerokostas, Gerasimos Kontogeorgis, Alexandros Leroutsos, Vasileios Papadoulis, Aikaterini Simou, Athanasios Chasiotis, Ioannis Thanasas

**Affiliations:** 1 Department of Health Sciences, Medical School, Aristotle University of Thessaloniki, Thessaloniki, GRC; 2 Department of Obstetrics and Gynecology, General Hospital of Trikala, Trikala, GRC; 3 Department of Anesthesiology, General Hospital of Trikala, Trikala, GRC; 4 Department of Obstetrics and Gynecology, University of Thessaly, School of Health Sciences, Larisa, GRC

**Keywords:** vasa previa, prenatal diagnosis, ultrasound, transvaginal doppler ultrasound, management, prognosis, case report

## Abstract

Vasa previa is a rare disorder of the placenta. The absence of a prenatal diagnosis is associated with increased perinatal morbidity and mortality. In our patient, ultrasound findings, although atypical, successfully established the prenatal diagnosis of vasa previa in the second trimester of pregnancy. Despite the fact that the placenta was not low-lying, that it was not possible to visualize the site of umbilical cord insertion into the placental tissue, and that vasa previa was not directly visualized, the presence of blood flow near and around the internal cervical os, as seen on transvaginal Doppler ultrasound in the second and third trimesters of pregnancy, raised serious suspicion of their presence. With the completion of the 36th gestational week, it was decided to proceed with a scheduled cesarean section. One week earlier, a course of corticosteroids was administered. The cesarean section was performed without complications. After placental delivery, the presence of velamentous umbilical cord insertion was noted, with umbilical vessels coursing unprotected by the placental tissue or umbilical cord within the fetal membranes. The puerperant and the newborn were discharged from the obstetrics clinic of the General Hospital of Trikala in excellent condition. This paper highlights the importance of transvaginal color Doppler ultrasound in the prenatal diagnosis of vasa previa, which, while posing little risk to the mother, can often be fatal to the fetus.

## Introduction

Vasa previa refers to arterial or venous fetal vessels that are not protected by the placental tissue or umbilical cord, coursing over the fetal membranes and located between the internal cervical os and the presenting part of the fetus [1]. Three types of vasa previa have been described to date (type I, type II, and type III). In type I vasa previa (as in our case), the umbilical cord arises from the fetal membranes (velamentous umbilical cord insertion). The umbilical vessels within the fetal membranes pass in front of the internal cervical os and enter the placenta [2]. Type II vasa previa requires the presence of a bilobed placenta. The fetal umbilical vessels course unprotected within the fetal membranes between the two placental lobes in front of the internal cervical os [3]. In type III vasa previa, the umbilical cord normally enters the placenta (central or peripheral insertion of the umbilical cord), but the umbilical vessels extend from one end of the placenta to the other, coursing within the fetal membranes [4].

Vasa previa is a rare placental disorder, the pathogenesis of which has not yet been fully clarified. In vitro fertilization techniques, multiple pregnancies, previous cesarean sections, a low-lying placenta, the presence of a bilobed placenta, and velamentous umbilical cord insertion are the main predisposing factors, estimated to account for the majority of cases (85%) and the increased incidence of the condition reported in recent years [5]. Overall, the incidence of vasa previa is estimated to be approximately one in 1,000 to one in 2,500 births [2,6,7]. The most common type of vasa previa is type I. Type II vasa previa accounts for approximately 21.3% of all cases [3]. The presence of placenta previa associated with velamentous cord insertion is seen in the majority of cases of type I vasa previa [8]. Furthermore, the incidence of type I vasa previa seems to increase significantly in women who conceive via in vitro fertilization. It is estimated that women who conceive using assisted reproductive techniques more frequently develop velamentous umbilical cord insertion compared to those who conceive spontaneously [2,9].

This paper highlights the rarity of vasa previa, which poses little risk to the mother but can often be fatal to the fetus. It also emphasizes the importance of transvaginal color Doppler ultrasonography in the prenatal diagnosis of vasa previa, which is estimated to significantly reduce the rate of perinatal morbidity and mortality associated with this condition.

## Case presentation

**Figure 1:**
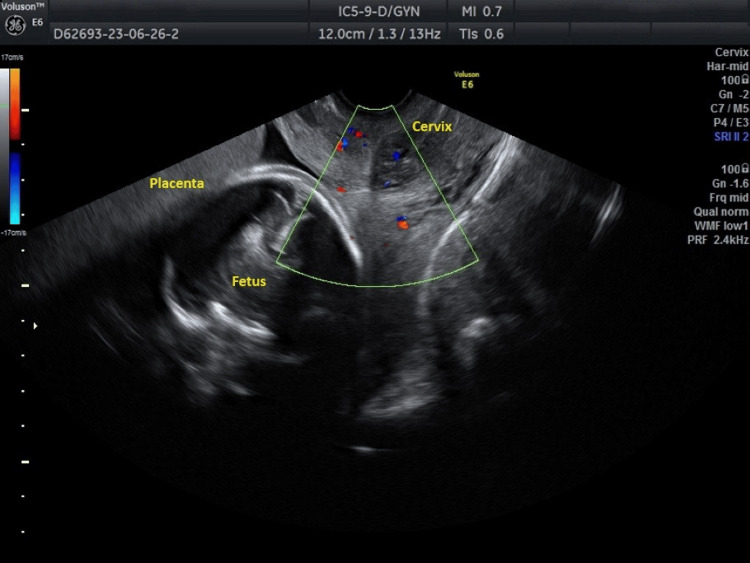
Transvaginal Doppler ultrasound imaging suggesting vasa previa during the second trimester of pregnancy The presence of blood flow near and around the internal cervical os of the uterus raises strong suspicion for the presence of vasa previa.

A 26-year-old primigravida, in her 32nd gestational week, was referred from a private prenatal care center in Trikala to the obstetrics clinic of the General Hospital of Trikala. The patient reported no pre-existing medical conditions, and her pregnancy has been progressing without any significant issues. The pregnancy occurred spontaneously. The referral was prompted by a concerning finding during a prenatal ultrasound examination, suggesting the possibility of vasa previa. During the second trimester ultrasound at 22 gestational weeks, blood flow was observed close to the internal cervical os of the uterus, raising suspicion for vasa previa (Figure 1).

**Figure 2:**
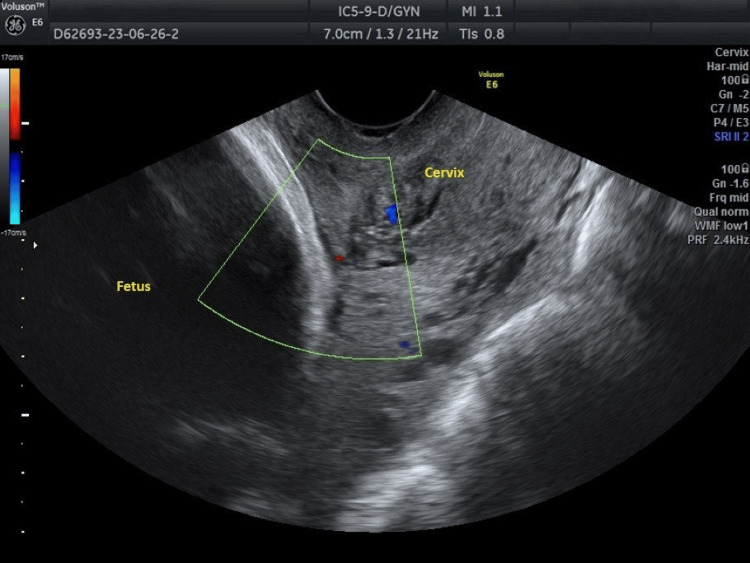
Transvaginal Doppler ultrasound imaging of the vasa previa during the third trimester of pregnancy Persistent blood flow near and around the internal cervical os further reinforces the suspicion of the presence of vasa previa.

However, definitive confirmation of vasa previa was not achieved at this time. Subsequent ultrasound imaging during the third trimester at 31 gestational weeks also detected blood flow near the internal cervical os, consistent with the previous findings (Figure 2).

Despite repeated examinations, clear visualization of the vasa previa, or the site of umbilical cord insertion into the placenta, was not achieved. Additionally, the anatomy of the placenta, such as whether it was single or bilobed, remained indistinct.

**Figure 3:**
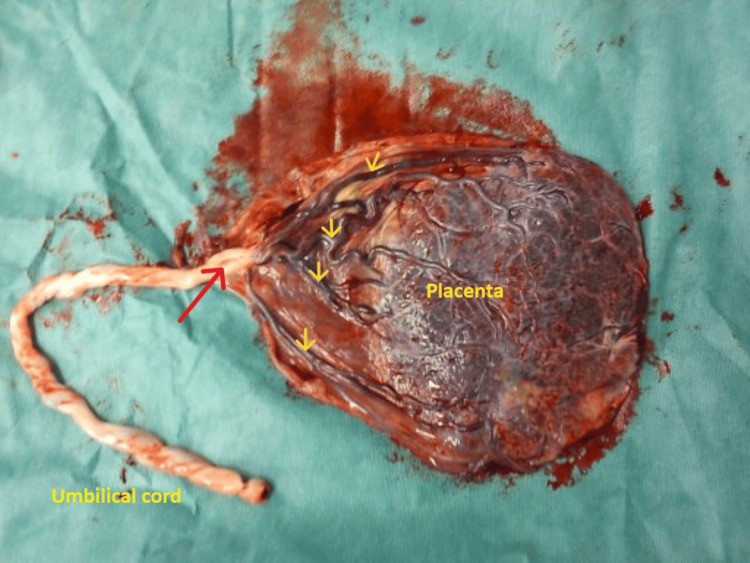
Type I vasa previa Velamentous umbilical cord insertion (red arrow) and the presence of fetal umbilical vessels that are not protected by the placental tissue or umbilical cord within the fetal membranes (yellow arrows) are evident.

Based on the atypical ultrasound findings described above, a diagnosis of vasa previa was established, leading to the decision to schedule a cesarean section for the 36th gestational week. The patient remained asymptomatic, and preventive hospitalization was deemed unnecessary. Corticosteroids (four doses of 6 mg dexamethasone, administered intramuscularly with a 12-hour interval between each dose) were prescribed one week prior to the scheduled cesarean section to promote fetal lung maturation. The cesarean section proceeded without complications, and blood loss was within normal limits. Following umbilical cord ligation and placental delivery, velamentous umbilical cord insertion was observed, characterized by the fetal umbilical vessels coursing unprotected within the fetal membranes (Figure 3).

Given the favorable condition of the neonate, weighing 2,580 g, admission to the neonatal intensive care unit (NICU) was deemed unnecessary. Following five days of observation and care in the obstetrics clinic of the General Hospital of Trikala, both the puerperant and the newborn were discharged in good general condition. 

## Discussion

Prenatal diagnosis of vasa previa presents significant challenges. However, it is essential for preventing perinatal morbidity and mortality [9]. Clinical diagnosis of vasa previa may sometimes occur incidentally in cases where pulses from the unprotected fetal vessels can be palpated following the onset of preterm labor or labor at term gestation without rupture of the fetal membranes [10]. Prior to the widespread availability of ultrasound in routine obstetric clinical practice, vasa previa was typically initially identified through sudden and significant vaginal bleeding, coupled with the inability to detect the fetal pulse due to spontaneous rupture of fetal membranes [11]. Surprisingly, none of the primary risk factors for vasa previa were identified during the prenatal period in our patient. The pregnancy was a singleton and resulted from spontaneous conception. In our patient, there was no history of previous cesarean sections, and neither placenta previa nor bilobed placenta were detected. Additionally, ultrasound imaging of velamentous umbilical cord insertion was not feasible. The diagnosis of vasa previa was established solely based on atypical ultrasonographic findings obtained during transvaginal color Doppler ultrasonography in the second trimester of pregnancy. This facilitated early and evidence-based management of this rare obstetric complication, aiming to reduce perinatal morbidity and mortality during pregnancy and delivery [12].

In contemporary obstetrics, transvaginal ultrasound combined with color Doppler ultrasound is recognized as the most effective tool for early and accurate prenatal diagnosis of vasa previa [13]. A key indicator of vasa previa on color transvaginal Doppler ultrasonography is the presence of linear or circular vascular structures above the cervix, corresponding to fetal vessels covering the internal cervical os [14]. During the second-trimester ultrasound, routine screening for identification of umbilical cord insertion to the placenta and increased vascularization over the internal cervical os should be conducted for all pregnant women. Additionally, screening for increased vascularization above the cervix should be repeated using transvaginal Doppler ultrasound at 32 gestational weeks. It has been noted that the diagnostic accuracy of vasa previa significantly improves in pregnant women diagnosed with placenta previa in the second trimester of gestation [1,15]. In our patient, a thorough examination of the cervix using transvaginal color Doppler ultrasound in the second and third trimesters of pregnancy did not reveal the typical presence of linear or circular vascular structures close to the inner cervical os of the uterus. Despite obtaining various ultrasound images by rotating the ultrasound probe to visualize the cervix both longitudinally and transversely, none of the images depicted the typical vascular structures over the cervix. The prenatal diagnosis of vasa previa in the second trimester of pregnancy in our patient was established based on the presence of blood flow near and around the inner cervical os of the uterus, without concurrent placenta previa (Figures 1-2).

The utilization of MRI during pregnancy has expanded beyond its established role in fetal imaging to include accurate assessment of the placenta [16]. Magnetic resonance imaging is now recognized as a valuable tool in diagnosing vasa previa, particularly in cases where suspected vessels near the internal cervical os cannot be adequately evaluated via transvaginal Doppler ultrasound [17]. Moreover, non-contrast time-of-flight magnetic resonance angiography may serve as an alternative for prenatal diagnosis and three-dimensional vascular assessment of vasa previa [18]. Despite these advancements, MRI was not utilized in our patient's case. The primary reason for this decision was the lack of experience among the physicians in the radiology department of our hospital, which precluded the performance of the imaging test necessary to determine the type of vasa previa. It is believed that identifying the type of vasa previa can significantly enhance the clinical evaluation and surgical management strategy for these patients, ultimately aiming to reduce perinatal morbidity and mortality [19].

The management of pregnant women diagnosed with vasa previa depends on the timing of the diagnosis and the presence or absence of symptoms. Prenatal identification of vasa previa mandates scheduling a cesarean section [20]. In asymptomatic pregnant women, a scheduled cesarean section should be conducted before the onset of labor or rupture of fetal membranes, ideally between 34 and 37 gestational weeks [21]. An emergency cesarean section becomes necessary if an undiagnosed vasa previa ruptures following the spontaneous onset of labor to safeguard the fetus's life [22]. During a cesarean section, it is crucial to avoid dissecting the fetal vessels to minimize fetal blood loss and the need for neonatal transfusion [23]. Administration of corticosteroids seven days before the procedure may be considered for pregnancies under 37 gestational weeks [24], though recent evidence suggests that routine corticosteroid administration in patients with vasa previa may not be warranted. Instead, risk assessment and corticosteroid administration should be planned within seven days of anticipated delivery [1]. After discussing this with our patient, we decided to administer prenatal corticosteroids. This decision was influenced by the fact that our hospital lacks a NICU for premature newborns, aiming to avoid the need for transfer and hospitalization in a tertiary facility. Additionally, the absence of risk factors in our patient led to the decision not to hospitalize her. Asymptomatic pregnant women with vasa previa and no risk factors can be managed as outpatients, provided that immediate access to the hospital is ensured in case of bleeding [25]. However, preventive maternal hospitalization and early termination of pregnancy by cesarean section should be considered for symptomatic patients and those at risk of preterm delivery [26,27]. Lastly, laser embryoscopic ablation before 33 gestational weeks, an experimental-stage treatment option for type II and type III vasa previa, may enable vaginal delivery [1,28].

The prognosis for the pregnant woman is generally favorable. However, the prognosis for the fetus depends largely on timely diagnosis and the extent of fetal blood loss, with reported cases of intrauterine deaths associated with vasa previa even in the absence of bleeding [29]. Cases diagnosed before 24 gestational weeks typically have an excellent prognosis. Prenatal diagnosis of vasa previa is usually not linked with perinatal fetal mortality [30,31]. Conversely, in pregnant women with undiagnosed vasa previa, fetal mortality rates significantly rise, with estimates suggesting they can be as high as 44% [22,32].

## Conclusions

Vasa previa is a rare and serious complication of pregnancy, potentially life-threatening for the fetus. Due to the rarity of the condition, there are no randomized controlled trials to guide the diagnosis and management of these patients. The contribution of transvaginal color Doppler ultrasound to the prenatal diagnosis of vasa previa is significant. Ultrasound findings, even when they are atypical and do not meet the typical published diagnostic criteria for vasa previa, should be taken seriously with the aim of early prenatal diagnosis of the condition and reduction of perinatal morbidity and mortality.
